# A Case Report of Sphenopalatine Ganglion Block Relieving Chronic Pain Post-Dental Bone Graft Surgery

**DOI:** 10.7759/cureus.45266

**Published:** 2023-09-14

**Authors:** Danielle Levin, Teddy Gerges, Martin Acquadro

**Affiliations:** 1 Anesthesiology, St. Elizabeth's Medical Center, Brighton, USA

**Keywords:** persistent idiopathic facial pain syndrome, bone graft, orofacial pain, chronic pain, sphenopalatine ganglion block

## Abstract

Chronic orofacial pain, by definition, is a pain that can anatomically extend anywhere between the area just under the orbitomeatal line, anterior to the pinnae, and above the neck. It occurs for 15 days or more per month, lasting four or more hours daily, for at least three months. Chronic orofacial pain, including persistent idiopathic facial pain syndrome, can significantly impact patients' quality of life and pose challenges for effective management. This case report describes a successful transnasal approach in treating a patient with severe oral pain following a bone graft surgery by blocking the sphenopalatine ganglion. The block provided significant pain relief and improved the patient's daily functioning. This minimally invasive treatment option offers an alternative for managing chronic orofacial pain after dental procedures such as bone graft surgery.

## Introduction

Chronic orofacial pain is a prevalent condition that can significantly impact patients' well-being [1]. When this pain occurs with no objectively identifiable neurological deficits and persists, it is often classified as persistent idiopathic facial pain syndrome [2]. Managing this type of pain can be complex due to the lack of standardized treatment approaches [2]. Traditional medication management similar to the management of other painful neuropathies may not always provide effective relief and can be associated with complications such as drug interactions and side effects [3,4]. Therefore, exploring minimally invasive treatment options becomes crucial.

One such option is the transnasal sphenopalatine ganglionic block, which has demonstrated effectiveness in various painful conditions, including headaches, neck pains, back pains, and post-operative shoulder pains [5-10]. The transnasal sphenopalatine ganglion block is a valuable and straightforward technique that was first discovered by Dr. Sluder over a century ago [11]. However, even today, only a limited number of healthcare providers are familiar with this type of block. Dr. Sluder initially performed the block by administering a 20% cocaine solution into the sphenopalatine region in 1908. In 1985, Dr. Kittrelle demonstrated that lidocaine 4% solution had an equivalent potency to cocaine for performing this block. Subsequently, few healthcare companies have developed intranasal devices for the administration of the transnasal sphenopalatine ganglion block [12]; however, these devices can be costly and may not be readily available in all healthcare centers.

The sphenopalatine ganglion is a bilateral collection of extracranial sympathetic, parasympathetic, and sensory neurons. It is the largest ganglion of its kind [12]. The axons originating from this ganglion transmit nerve signals and influence local blood flow to the nasal and lacrimal glands [11,12]. Activation of the sphenopalatine ganglion leads to cerebral vasodilation through the release of acetylcholine, vasoactive intestinal peptide, and nitric oxide. This increased flow of chemicals can lead to plasma protein extravasation and contribute to the development of chronic pain. By administering a sphenopalatine ganglion block, the neurons in this area are effectively blocked, potentially disrupting the cycle of chronic pain, and a possible reset occurs [13]. The ganglion is covered by a thin layer of connective tissue, approximately 1-2 mm thick, making it accessible for topical administration of medication [13].

Our sphenopalatine ganglion block technique involves the application of lidocaine 4% topical solution on cotton-tipped applicators, which are gently inserted intranasally for 15-minute intervals [13]. The local anesthetic solution drips from the nose into the back of the throat, and as it drips through this path, the sphenopalatine ganglion gets coated with the local anesthetic because it is in that pathway. By targeting the sphenopalatine ganglion, this block can provide substantial pain relief via a minimally invasive approach and practically no side effects. Contraindications to sphenopalatine ganglion block include allergy to any of the medications used, anticoagulation, history of facial trauma, infection, and patient refusal.

In this report, we present a case of a patient with persistent idiopathic facial pain syndrome who experienced short-term but consistent pain relief exceeding 80% following transnasal sphenopalatine ganglion blocks. Written informed consent was obtained from the patient to share this report.

## Case presentation

History of chief complaint

A 76-year-old female presented to the pain clinic with chronic, severe oral pain. The patient had been edentulous for more than 20 years and had previously experienced no issues with her dentures. However, in recent years, she required multiple adjustments to her dentures and eventually underwent bone graft surgery in preparation for permanent upper implants. Following the bone grafts, her dentist and oral surgeon confirmed proper healing. The patient did not recall any specific incidence of pain onset. The pain was located on the upper palate, posterior to the incisors, bilaterally, and was exacerbated by eating or manipulating the mouth while wearing dentures. Removing the dentures and abstaining from eating or talking provided slight relief. The patient had been suffering from this pain for nine months, leading to functional limitations in daily activities, disrupted sleep, mood disturbances, and socialization difficulties. On physical examination, no visual abnormalities were evident.

Past treatment

Before seeking treatment at our pain clinic, the patient tried various approaches without success. These included over-the-counter gels containing benzocaine and menthol, as well as prescribed medications such as gabapentin, promethazine, and amitriptyline.

Current treatment

Considering previous reports in the literature regarding the effectiveness of the sphenopalatine ganglion block for relieving pain of different origins [5-10,13], we proposed this treatment option to the patient. After discussing the risks and benefits of the procedure, the patient provided written informed consent.

**Figure 1:**
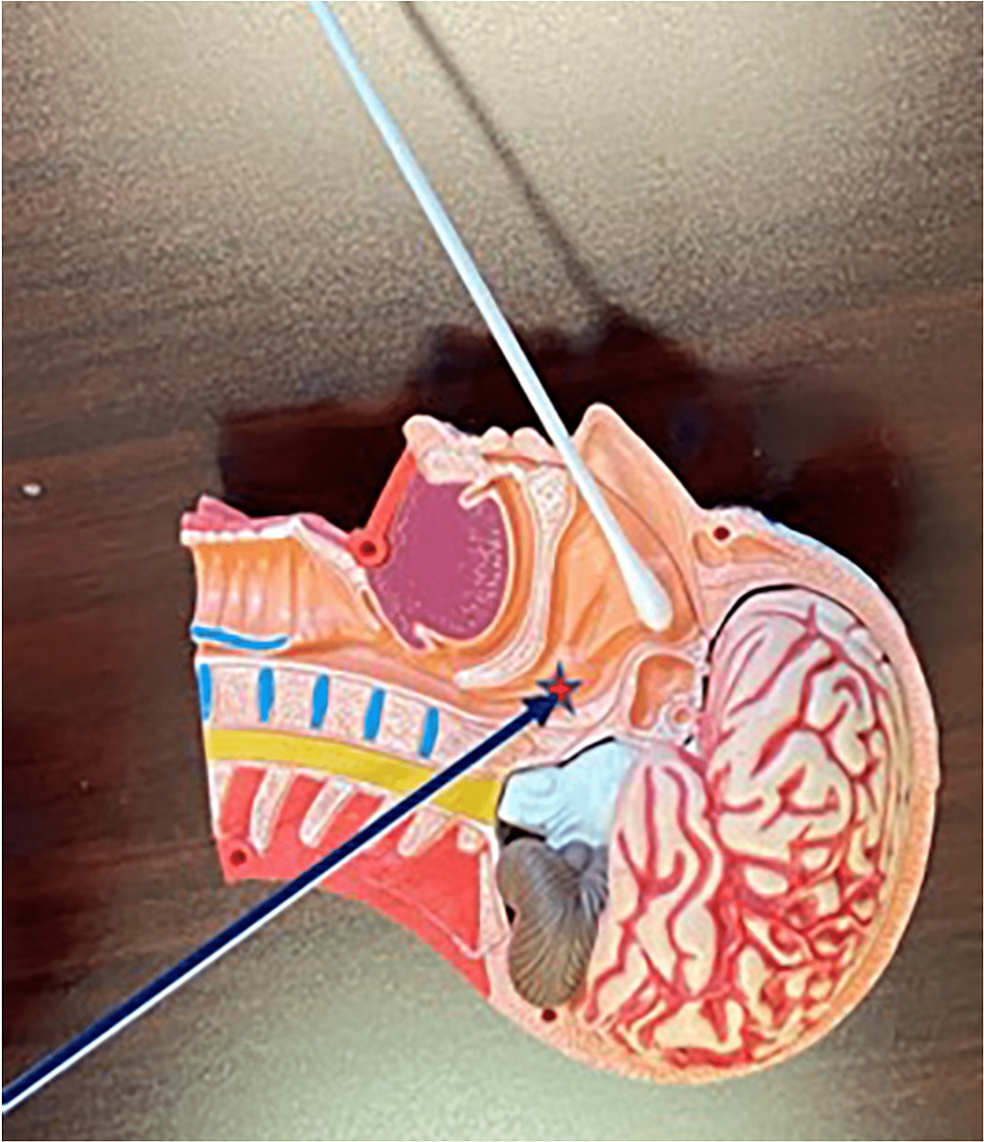
Mannequin model of the sphenopalatine ganglion block The sphenopalatine ganglion applicator on a mannequin model demonstrating that once the patient feels the medication in the back of the throat, the medication has reached the sphenopalatine ganglion (marked with the red star). Image Credits: Danielle Levin, MD.

During the procedure, the patient was positioned in a supine position, with their head tilted slightly back, and hemodynamics were monitored using a non-invasive blood pressure cuff and pulse oximeter [11]. Long cotton-tipped applicators were soaked in 4% lidocaine topical solution (preservative-free) and inserted one at a time into each nostril aiming toward the back of the nasal cavity (Figure 1).

**Figure 2:**
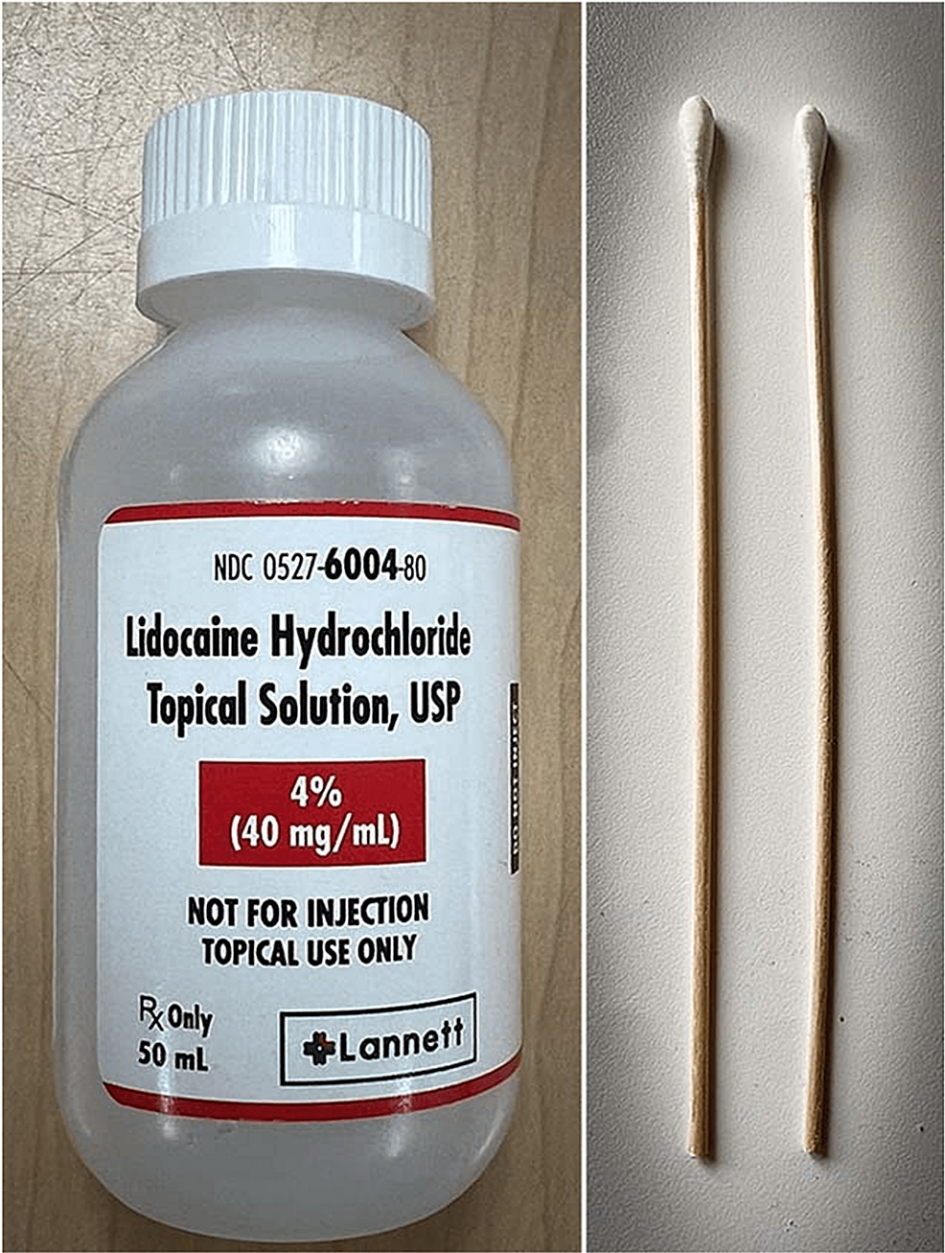
Setting up the sphenopalatine ganglion block applicator. Image Credits: Danielle Levin, MD.

The cotton tips were gently advanced until a slight resistance was met. The applicators were left in place for 15 minutes allowing time for local lidocaine diffusion, and then the exact process was performed twice, totaling 45 minutes (Figure 2). After each 15-minute application, the patient reported a gradual pain reduction, and after the last 15-minute application, the patient was completely pain-free.

The cotton-tip applicators were removed intact, and the patient was monitored for an additional 10 minutes before being discharged home. The patient remained hemodynamically stable throughout the procedure and experienced no side effects. Prior to leaving, the patient continued to be completely pain-free.

At the one-month follow-up visit, the patient reported experiencing more than 80% pain relief for a period exceeding two weeks after the treatment. She denied having any adverse reactions from the procedure and observed significant improvement in her daily functioning. However, the pain gradually started to return back. During this follow-up visit, the patient received another sphenopalatine ganglion block administered following the same technique. Immediately after the block, the patient was free of pain. During the block, the patient remained hemodynamically stable, without any side effects. She experienced more than 80% pain relief for four days following this treatment.

One month after the second sphenopalatine ganglion block, the patient underwent a third procedure. Similar to the previous instances, the patient tolerated the procedure with no complications, achieved complete pain relief following the block, and experienced more than 80% pain relief for four days post-treatment. Then, the patient's pain slowly returned to pre-treatment level.

## Discussion

Grafting bone fragments is a widely performed procedure in modern medicine and dentistry. The history of successful bone grafting dates back to 1668 when Dr. Job van Meekeren of Amsterdam documented the repair of a soldier's skull defect using a bone graft from a dog, yielding positive results. With the advancements in anesthetics and the implementation of antisepsis, bone grafting became a feasible clinical procedure with minimal postoperative discomfort [13,14].

Pain following surgical procedures can stem from various processes, including nociceptive, inflammatory, neuropathic, and nociplastic mechanisms [15]. Nociceptive and inflammatory pain are integral parts of the healing process, triggered by tissue damage. Nociceptive pain manifests as sharp, aching, or throbbing sensations following an acute noxious stimulus. If the noxious stimulus persists, nociceptive neurons release pro-inflammatory markers, leading to the transition from acute pain to inflammatory pain. The inflammatory process is a normal biological response to tissue injury and serves a protective function for the patient. Typically, nociceptive or inflammatory pain is short-lasting compared to nociplastic pain, which arises from altered nociception without clear evidence of actual or threatened tissue damage, resulting in the activation of peripheral nociceptors and prolonged pain. When nerve injuries occur during or after surgical procedures it can contribute to prolonged or chronic pain. In fact, when the somatosensory nervous system is affected, neuropathic pain emerges. Clinically, neuropathic pain presents as spontaneous, ongoing, or shooting pain that is exacerbated by both noxious and non-noxious stimuli [16,17].

In addition to neuropathic pain, once any pain condition becomes chronic, remodeling occurs in the brain causing central sensitization with accompanying allodynia (non-painful stimulus being perceived as excruciating pain) and hyperalgesia (mildly painful stimulus being perceived as excruciating pain). This could have been another etiology of the patient's pain.

According to the International Classification of Orofacial Pain, persistent idiopathic facial pain syndrome is a chronic pain condition characterized by unremitting pain in the face and/or oral area, without identifiable structural correlates. This condition often arises following invasive procedures and shares similarities with neuropathic pain, warranting similar management approaches [2]. Our patient likely had persistent idiopathic facial pain syndrome given her nine-month history of severe orofacial pain secondary to a traumatic neuropathic mechanism following a dental bone graft surgery.

Following Bayer et al.'s observational study, some authors have suggested pulsed radiofrequency treatment of the sphenopalatine ganglion as a potential option for managing persistent idiopathic facial pain syndrome when other treatments prove ineffective [3,18]. In our patient’s case, conservative management and pharmacological treatments, including gabapentin, promethazine, amitriptyline, and topical benzocaine, failed to provide sufficient pain relief. Therefore, we offered an alternative to the pulsed radiofrequency treatment involving a minimally invasive transnasal sphenopalatine ganglion block. This approach, administered through lidocaine drops and a cotton-tipped applicator, proved highly effective for our patient, offering a less invasive alternative to the traditional pulsed radiofrequency treatment. However, our patient only experienced a short-term pain relief with our treatment. To extend pain relief for patients like ours, we can consider teaching patients to administer the block by themselves at home so that they do not have to come to the office often for frequent administrations. Also, the in-office sphenopalatine ganglion block can be considered as a test block if the effect of the block is short-lived, and then the pulsed radiofrequency treatment can be considered.

In our pain clinic, we prefer to administer the transnasal sphenopalatine ganglion block using supplies that are commonly found in most clinics. We perform the block using long cotton-tipped applicators soaked in lidocaine 4% topical solution [13]. In our experience of performing the block for various pain conditions, all of our patients tolerated the procedure very well. One patient experienced mild nausea after she moved her head a lot during the procedure, but besides that patient, no other patients experienced any side effects, suggesting that this block is quite safe.

## Conclusions

In conclusion, the transnasal sphenopalatine ganglion block proved to be an effective short-term treatment for persistent idiopathic facial pain syndrome following dental bone graft surgery in this case. This treatment option is characterized by its simplicity, safety, and cost-effectiveness, providing significant pain relief beyond the temporary effects of local anesthetics. Healthcare providers should consider the transnasal sphenopalatine ganglion block as a viable alternative for managing chronic orofacial pain in patients with a history of dental procedures. Also, healthcare providers can consider teaching patients how to perform this block by themselves at home. Further research and increased awareness of this approach are necessary to enhance pain management outcomes and expand the block's utilization.
